# The CaMK4/CREB/IRS-2 Cascade Stimulates Proliferation and Inhibits Apoptosis of β-Cells

**DOI:** 10.1371/journal.pone.0045711

**Published:** 2012-09-25

**Authors:** Bo Liu, Helena Barbosa-Sampaio, Peter M. Jones, Shanta J. Persaud, Dany S. Muller

**Affiliations:** Diabetes Research Group, School of Medicine, Division of Diabetes & Nutritional Sciences, King’s College London, London, United Kingdom; National Center for Scientific Research Demokritos, Greece

## Abstract

Progressive reduction in β-cell mass is responsible for the development of type 2 diabetes mellitus, and alteration in insulin receptor substrate 2 (IRS-2) abundance plays a critical role in this process. IRS-2 expression is stimulated by the transcription factor cAMP response element-binding protein (CREB) and we recently demonstrated that Ca^2+^/calmodulin dependent kinase 4 (CaMK4) is upstream of CREB activation in β-cells. This study investigated whether CaMK4 is also a potential target to increase β-cell mass through CREB-mediated IRS-2 expression, by quantifying mouse MIN6 β-cell proliferation and apoptosis following IRS-2 knockdown, CaMKs inhibition and alterations in CaMK4 and CREB expression. Expression of constitutively active CaMK4 (ΔCaMK4) and CREB (CREB_DIEDLM_) significantly stimulated β-cell proliferation and survival. In contrast, expression of their corresponding dominant negative forms (Δ^K75E^CaMK4 and CREB_M1_) and silencing of IRS-2 increased apoptosis and reduced β-cell division. Moreover, CREB_DIEDLM_ and CREB_M1_ expression completely abolished the effects of Δ^K75E^CaMK4 and of ΔCaMK4, respectively. Our results indicate that CaMK4 regulates β-cell proliferation and apoptosis in a CREB-dependent manner and that CaMK4-induced IRS-2 expression is important in these processes.

## Introduction

Type 2 diabetes mellitus (T2DM) is a metabolic disorder caused by a progressive decline in β-cell function [1] and β-cell mass [2], [3]. It usually occurs following the development of insulin resistance [4], [5], but this may not be directly responsible for the development of T2DM since most obese people who have mild to severe insulin resistance do not develop the disease [2], [3], [6] due to a compensatory process involving increased β-cell function and β-cell mass expansion [2], [3], [7]. Similarly, the increased metabolic demand that is observed in pregnancy is compensated by an adaptive increase in β-cell mass [8], [9]. It is therefore possible that a defect in the mechanisms by which β-cell mass expansion occurs is responsible for development of the full phenotype of T2DM [10].

The control of β-cell mass plasticity involves a complex network of physiological processes that regulate the balance between β-cell proliferation/neogenesis [11]–[13] and apoptosis [14], [15]. Accordingly, stimulating proliferation/neogenesis and/or reducing apoptosis are direct ways of increasing β-cell mass in response to increased metabolic demand. Thus, identifying novel molecular β-cell targets that can be manipulated to promote proliferation and inhibit apoptosis has the potential for maintaining or expanding β-cell mass in T2DM.

*In vivo* experiments have demonstrated that mice deficient in insulin receptor substrate 2 (IRS-2) develop T2DM, in part due to a significant reduction in β-cell mass [16], [17], and that the targeted re-expression of IRS-2 in β-cells enhances their survival and promotes growth, mainly *via* increased proliferation [18]. Consistent with these observations, *in vitro* antisense-mediated decreased IRS-2 expression in INS-1 β-cells enhanced apoptosis [19], while IRS-2 over-expression in rodent and human islets was associated with a reduction in β-cell apoptosis [20]. Therefore, it is now well established that IRS-2 has a major role in the physiological processes that control β-cell mass plasticity and characterising the molecular mechanisms regulating its expression could lead to the development of novel therapies to treat T2DM.

Calcium/calmodulin-dependent kinase 4 (CaMK4) is a multifunctional serine/threonine protein kinase that was initially identified in the cerebellum, forebrain, testis, spleen and thymus [21], [22], in which its functions are best understood. It is activated in response to elevation in intracellular calcium by an upstream kinase, CaMK kinase (CaMKK) by phosphorylation of an activation loop threonine residue [23], and the CaMKK/CaMK4 cascade is reported to be involved in glucose-stimulated insulin promoter activation in INS-1 β-cells [24]. CaMK4 mediates calcium-dependent stimulation of dendritic growth, which is dependent on CaMK4-stimulated phosphorylation and activation of the transcription factor cAMP response element-binding protein (CREB) [25]. This CaMK4-CREB signalling cascade also inhibits apoptosis and promotes neuron and dendritic cell survival against various stresses [26]–[29]. In β-cells CaMK4 is activated by elevated glucose levels and increases in intracellular Ca^2+^ [24], [30] and we recently demonstrated that the CaMK4-CREB pathway mediates glucose stimulation of IRS-2 expression in mouse islets [31]. Thus, since both IRS-2 and CREB are known to increase β-cell proliferation and inhibit apoptosis, we hypothesise that CAMK4 has a central position in the processes by which β-cell mass is regulated. In this study we report that knockdown of IRS-2 reduces β-cell proliferation and increases β-cell apoptosis, while constitutively active CaMK4 and CREB stimulate β-cell proliferation and survival. In addition, the mass promoting effects of CaMK were abrogated by a dominant negative form of CREB. These studies therefore support a signalling cascade in which the CaMK4/CREB pathway is pivotal in β-cell mass regulation.

## Results

### IRS-2 Regulates MIN6 β-cell Proliferation and Apoptosis

**Figure 1:**
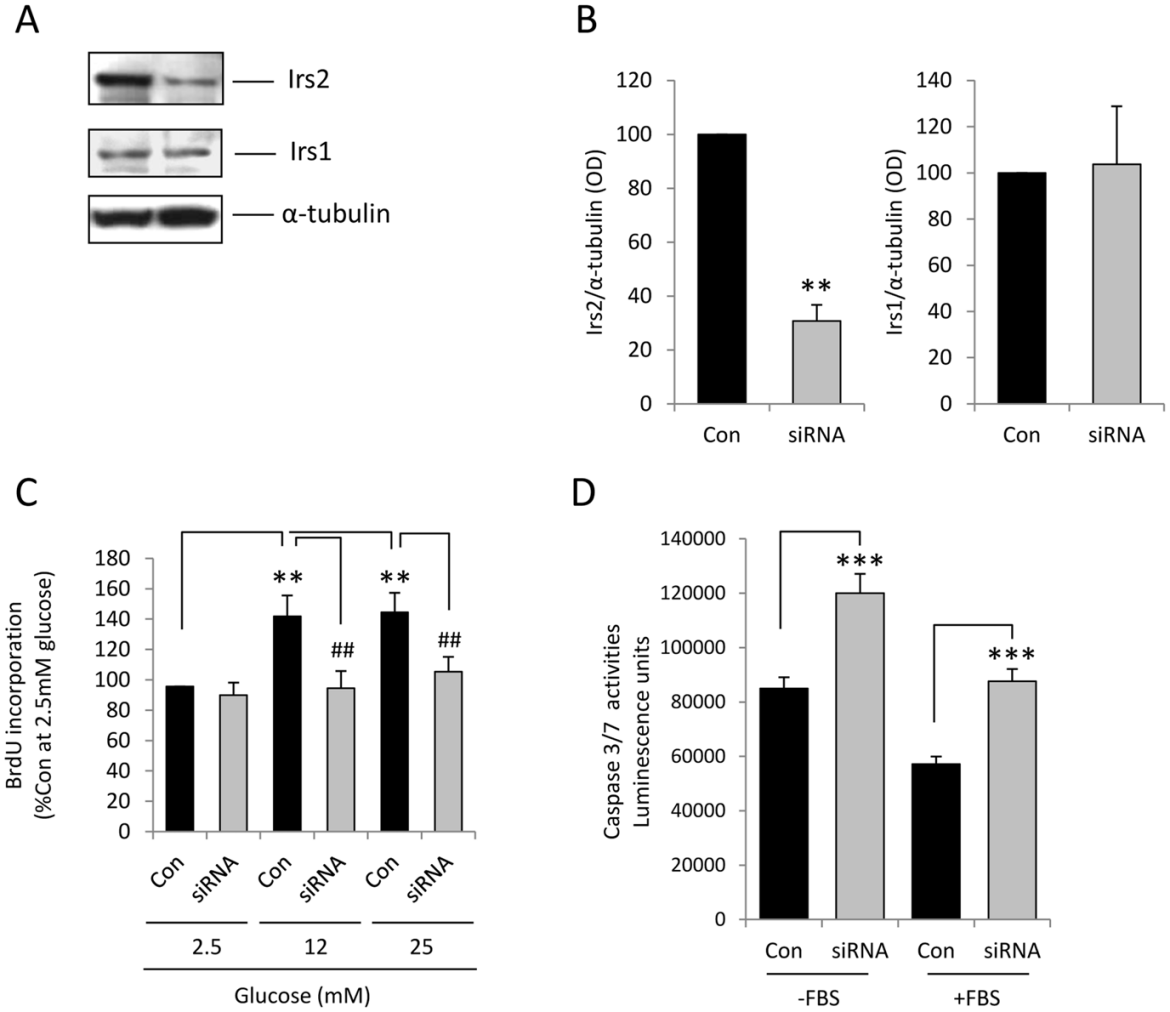
IRS-2 plays a pivotal role in the regulation of MIN6 β-cell proliferation and apoptosis. (**A**) IRS-2, IRS-1 and α-tubulin protein levels in MIN6 β-cells 24 h after transient transfection with 200 nM non-interfering RNA (left) or 200 nM siRNA (right) designed to knock down IRS-2. (**B**) Optical densitometry measurements of IRS-2 and IRS-1 quantified as ratios of α-tubulin expression levels obtained from Figure 1A. Data are means + SEM of 3 independent experiments, **p<0.01. (**C**) 24 h after transfection, MIN6 β-cells were maintained in culture for 48 h in the presence of 2.5–25mM glucose with 10% FBS and proliferation was determined by quantification of BrdU incorporation into newly synthesised DNA. The results are expressed as percentage of BrdU incorporation at 2.5mM glucose. Data are means + SEM, n = 3, **p<0.01 compared to the control samples at 2.5mM glucose and ^##^ *p*<0.01 compared to the control samples at 12 or 25mM glucose. (**D**) 24h after transfection, MIN6 β-cells were maintained in culture for 48h at 2.5 mM glucose in the absence of serum (-FBS) or in the presence of 10% FBS (+FBS) before caspase 3/7 activities were quantified. Data are means + SEM, n = 8, ***p<0.001 siRNA versus control; *p*<0.001–FBS versus +FBS controls.

Short term glucose administration has, to a certain extent, the capacity to stimulate β-cell proliferation and survival in rodents [32] and we have previously confirmed these observations in the mouse MIN6 β-cell line [33]. Therefore, to study the role of IRS-2 in the mechanisms by which β-cell proliferation and apoptosis are regulated its expression was knocked down in MIN6 β-cells by transient transfection with 200 nM small interfering RNA duplexes (siRNAs). The effects of 2.5–25 mM glucose on proliferation and apoptosis were assessed in these IRS-2-deficient cells and in MIN6 β-cells transiently transfected with 200 nM non-interfering RNA (niRNAs). As shown in Figures 1A and 1B Western blotting of protein-matched MIN6 β-cell extracts indicated that there was a 69.2±6.0% reduction in IRS-2 protein expression (*p*<0.01) when MIN6 β-cells were transfected with 200 nM of the IRS-2 siRNAs compared to control cells treated with niRNAs. The specificity of this knockdown effect was demonstrated by the observation that no significant alteration in insulin receptor substrate 1 (IRS-1) levels was detected between the niRNA- and siRNA-treated samples (Figure 1A–B).

The role of IRS-2 in the mechanisms by which glucose stimulates β-cell division was determined by measuring glucose-dependent BrdU incorporation into newly synthesised DNA in MIN6 β-cells transfected with IRS-2 siRNAs and niRNAs. As we have previously shown [33], glucose stimulated MIN6 β-cell proliferation in a concentration-dependent manner (Figure 1C, *p*<0.01) with a maximal effect at 12 mM glucose. In contrast, although no significant differences in basal proliferation were observed at 2.5 mM glucose between niRNA- and siRNA-treated cells, the stimulatory effect of glucose on β-cell division was significantly reduced following IRS-2 knockdown (*p*<0.01), suggesting a pivotal role for IRS-2 in glucose-induced MIN6 β-cell proliferation. In addition, caspase-3 and -7 activities were also significantly increased (*p*<0.001) following IRS-2 depletion, irrespective of whether the β-cells were maintained in the absence or presence of FBS. Thus, it can be seen from Figure 1D that caspase 3/7 activities were significantly higher (*p*<0.001) in control cells that had been maintained in the absence of FBS for 48 hours, as expected as serum withdrawal is an apoptosis-inducing stimulus. Nonetheless, there was an increase in apoptosis of over 40% in response to IRS-2 depletion in both the serum-maintained and serum-restricted groups of β-cells. These data are consistent with an anti-apoptotic role for IRS-2 under normal conditions and also when the cells were stressed by 48 hours of serum deprivation, and confirm previous *in vivo* and *in vitro* data suggesting that IRS-2 regulates β-cell survival [19], [20], [34], [35].

### CaMK4 Mediates the Glucose Stimulatory Effect on MIN6 β-cell Proliferation

**Figure 2:**
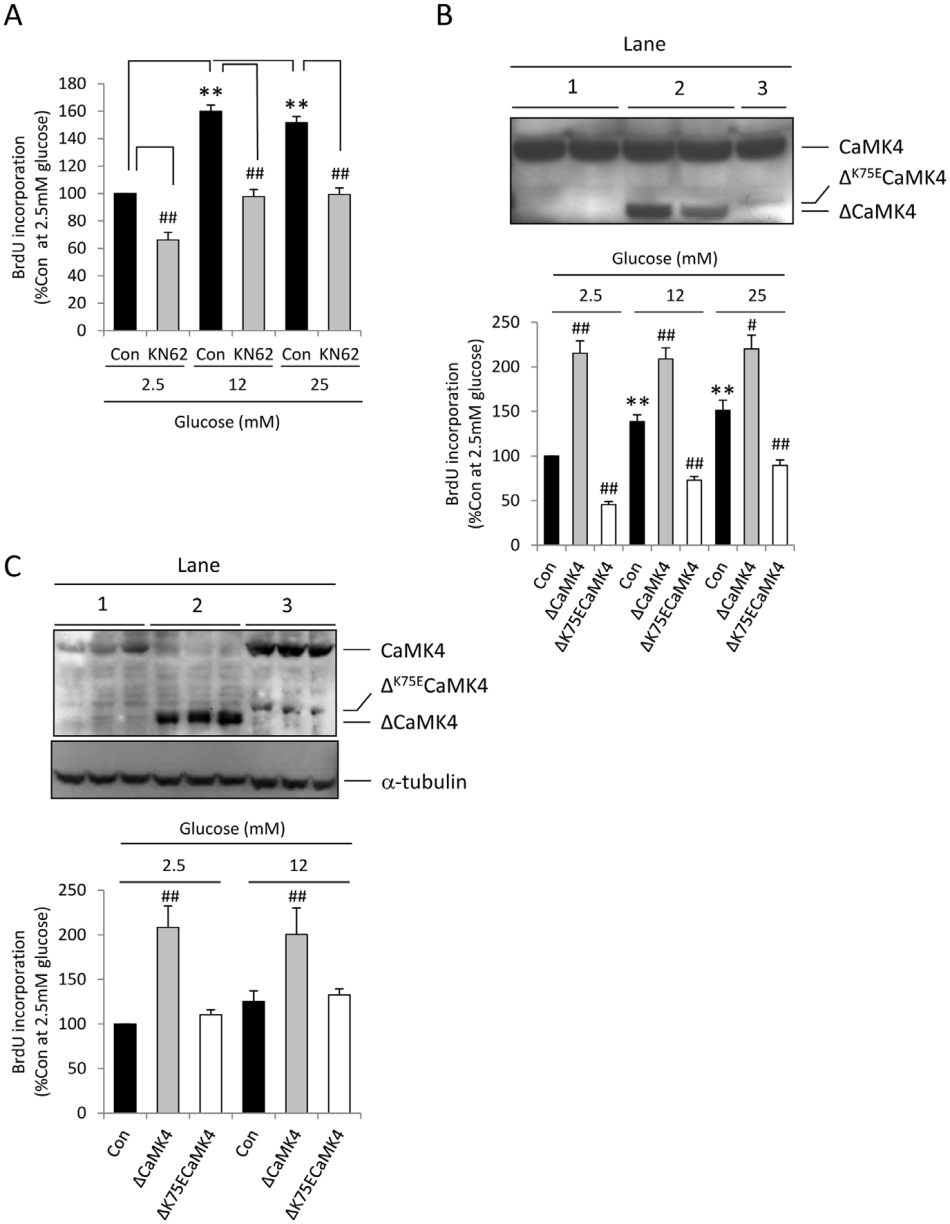
CaMK4 stimulates MIN6 β-cell proliferation. (**A**) Effect of the CaMKs inhibitor KN62 on glucose-stimulated proliferation. MIN6 β-cells were treated with 20µM KN62 or DMSO (Con) in the presence of 2.5–25 mM glucose for 48 h and proliferation was determined by quantification of BrdU incorporation into newly synthesised DNA. Data are means + SEM, n = 3, **p<0.01 compared to the control samples at 2.5 mM glucose and ^##^ *p*<0.01 compared to the control samples at 2.5, 12 or 25 mM glucose. (**B, C**) Effects of CaMK4 on MIN6 β-cell proliferation. (**B**) MIN6 β-cells were stably transfected with pcDNA3.1 (lane 1, Con), pcDNA3.1/ΔCaMK4 (lane 2, ΔCaMK4) and pcDNA3.1/Δ^K75E^CaMK4 (lane 3, Δ^K75E^CaMK4) and expression of endogenous CaMK4, ΔCaMK4 and Δ^K75E^CaMK4 was determined by Western blot once the colonies were established (upper panel). The transfectant MIN6 β-cells were then exposed to 2.5–25 mM glucose and cell proliferation was determined (lower panel). Results are means + SEM, n = 3. **p<0.01 compared to the control samples at 2.5 mM glucose. ^##^ *p*<0.01 compared to their relative control samples at 2.5, 12 or 25 mM glucose. (**C**) Endogenous CaMK4, ΔCaMK4 and Δ^K75E^CaMK4 expression levels and α-tubulin expression in the same samples (upper panel) and proliferation (lower panel) of the transfected MIN6 β-cells were determined again after 10–15 passages. Lanes 1, 2 and 3 are from cells transfected with pcDNA3.1, pcDNA3.1/ΔCaMK4 and pcDNA3.1/Δ^K75E^CaMK4, respectively. Results are means + SEM, n = 3. ^##^ *p*<0.01 compared to their relative control samples at 2.5 or 12 mM glucose.

Glucose is a potent stimulator of IRS-2 expression in β-cells [36], [37] and we recently demonstrated that this is dependent on Ca^2+^ influx, and CaMK4 mediates the stimulatory effect of glucose on IRS-2 [31]. Therefore, to study the role of CaMK4 in glucose-induced MIN6 β-cell proliferation, the effects of the non-selective CaMKs inhibitor KN62 on cell division were assessed. Figure 2A shows that the presence of 20 µM KN62, a concentration that we have previously shown inhibits glucose-stimulated CREB phosphorylation and IRS-2 up-regulation in MIN6 β-cells [31], significantly reduced BrdU incorporation at 2.5 mM glucose and suppressed the stimulatory effects of 12 and 25 mM glucose (*p*<0.01). These observations imply that one or more calcium/calmodulin-dependent kinase is involved in the signalling cascade through which glucose mediates its proliferative action.

The importance of CaMK4 in this process was investigated by generating MIN6 β-cell clones that stably expressed constitutively active ΔCaMK4, in which the autoregulatory domain had been deleted, or dominant negative Δ^K75E^CaMK4, which lacked the autoregulatory domain and the lysine involved in ATP binding had been replaced by glutamate [23]. It can be seen from Figure 2B (upper panel) that MIN6 β-cells transfected with an empty vector expressed the 60 kDa native CaMK4 alone (lane 1), whereas those cells containing mutant CaMK4 plasmids expressed both endogenous CaMK4 and a Ca^2+^/calmodulin-independent, truncated 35 kDa ΔCaMK4 protein (lane 2) or endogenous CaMK4 and a low level of the kinase dead 37 kDa Δ^K75E^CaMK4 protein (lane 3). As shown in Figure 2B (lower panel), proliferation was increased to its maximal at all glucose concentrations in ΔCaMK4-expressing MIN6 β-cells, whereas it was significantly reduced in Δ^K75E^CaMK4 transfectant cells. Interestingly, whereas the stimulatory effect of ΔCaMK4 was maintained after several passages, we observed a progressive loss in the inhibitory action of Δ^K75E^CaMK4 on proliferation (Figure 2C, lower panel). Therefore, the expression levels of the endogenous, dominant negative and constitutively active forms of CaMK4 were determined again by Western blot after a further 10–15 passages. The data showed that MIN6 β-cells compensated for the expression of Δ^K75E^CaMK4 by increasing expression of the functional, endogenous form of the kinase (Figure 2C, upper panel lane 3). A reduced exposure time was used for the blot shown in Figure 2C so that CaMK4 up-regulation in Δ^K75E^CaMK4 cells did not saturate the blot. Nonetheless, despite the low intensity of endogenous CaMK4 in the MIN6 β-cells transfected with an empty vector (Figure 2C, upper panel lane 1) it is readily visible, while endogenous CaMK4 was depleted following prolonged stable transfection of MIN6 β-cells with ΔCaMK4 (Figure 2C, upper panel lane 2).

### CREB Mediates the Stimulatory Effect of CaMK4 on MIN6 β-cell Proliferation

**Figure 3:**
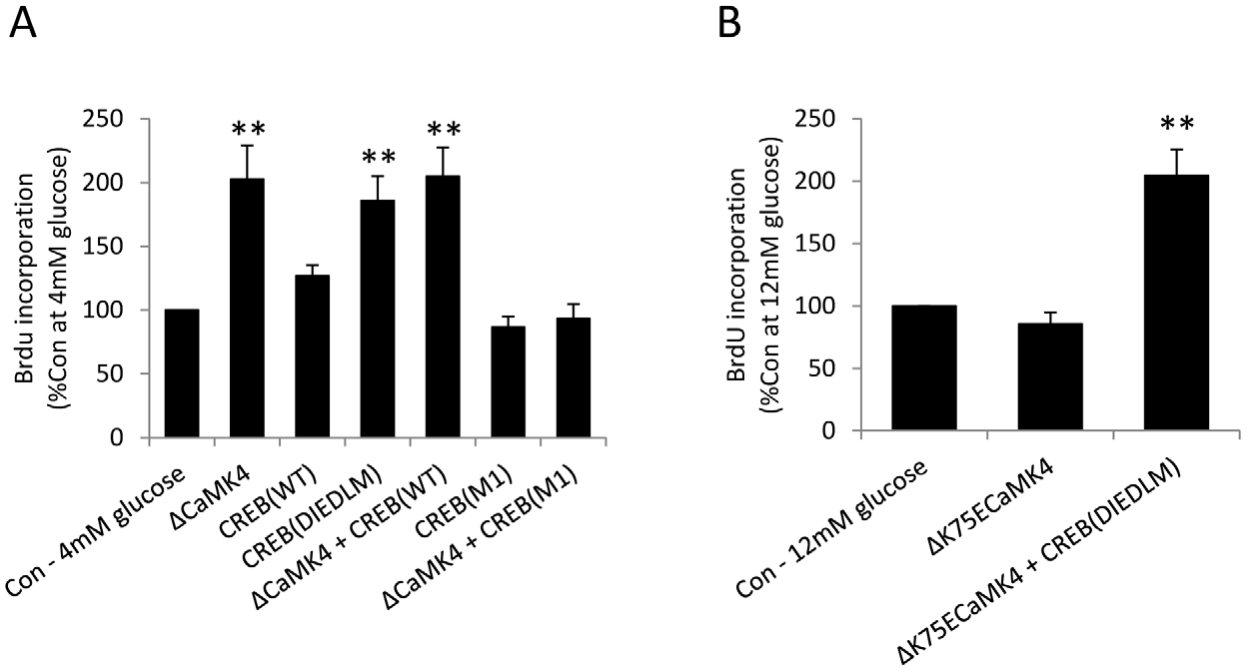
CaMK4 regulates MIN6 β-cell proliferation *via* CREB activation. (**A, B**) 24 h after MIN6 β-cells had been transiently transfected with plasmids as shown, they were maintained in culture for a further 48 h in the presence of 4 or 12 mM glucose and proliferation was determined by quantifying BrdU incorporation into newly synthesised DNA. Results are means + SEM, n = 3, **p<0.01 relative to the control samples at 4 mM or 12 mM glucose.

The transcription factor CREB is a major target of CaMK4 in neurons [25] and we have demonstrated that it is also phosphorylated following CaMK4 activation in islets [31]. The role of CREB in the molecular mechanisms by which CaMK4 stimulates β-cell proliferation was therefore assessed by transiently transfecting MIN6 β-cells with the CaMK4 mutants (Δ^K75E^CaMK4 or ΔCaMK4) in the absence or presence of wild-type (CREB_WT_), dominant negative (CREB_M1_) or constitutively active (CREB_DIEDLM_) forms of CREB [38]. Transfected β-cells were maintained in culture at 4 or 12 mM glucose for 48 hours and proliferation was quantified by BrdU incorporation. As already observed in stably transfected MIN6 β-cells (Figure 2C), proliferation was significantly increased (*p*<0.01) at a sub-stimulatory glucose concentration (4 mM) in cells expressing the constitutively active ΔCaMK4 form of the kinase (figure 3A), while there was a small reduction in glucose-stimulated proliferation in cells expressing Δ^K75E^CaMK4 (Figure 3B).

Expression of CREB_DIEDLM_, the constitutively active form of CREB, also significantly promoted MIN6 β-cell division (*p*<0.01) at 4 mM glucose (Figure 3A). However, neither CREB_M1_ nor CREB_WT_ overexpression significantly modified MIN6 β-cell proliferation and CREB_WT_ did not potentiate the stimulatory ΔCaMK4 effect at 4 mM glucose. In contrast, CREB_M1_ expression abolished the stimulatory action of ΔCaMK4 (Figure 3A, *p*<0.01). A stimulatory role for CREB downstream of CaMK4 was confirmed by an absence of inhibition of 12 mM glucose-induced BrdU incorporation following co-expression of Δ^K75E^CaMK4 and constitutively active CREB (CREB_DIEDLM_), and the extent of proliferation was significantly higher than in control cells at 12 mM glucose (Figure 3B, *p*<0.01).

### CaMK4 Promotes MIN6 β-cell Survival in a CREB-dependent Manner

**Figure 4:**
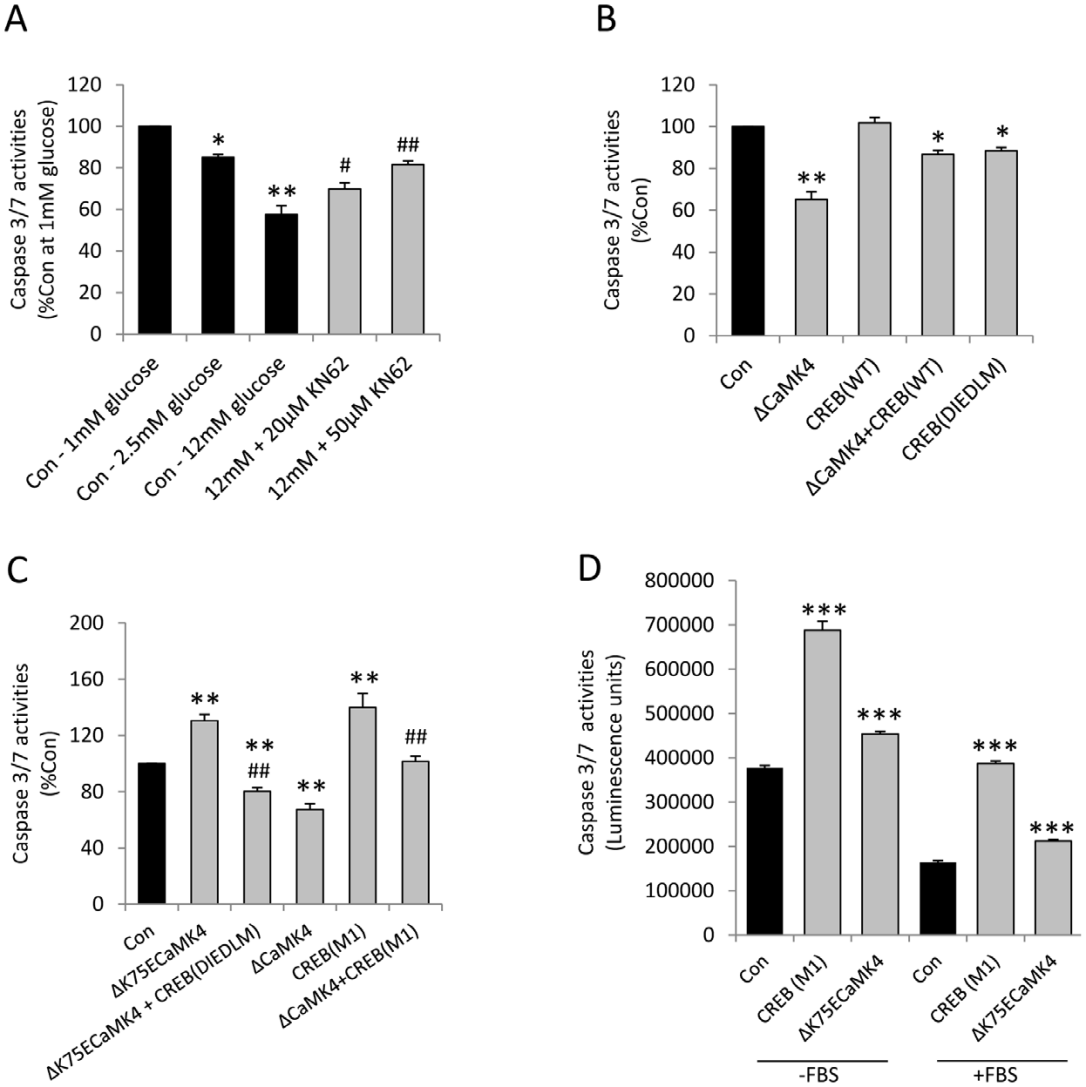
CaMK4 inhibits MIN6 β-cell apoptosis in a CREB-dependent manner. (**A**) Effect of the CaMKs inhibitor KN62 on MIN6 β-cell apoptosis. MIN6 β-cells were treated with 1–12 mM glucose and with 20–50 µM KN62 in the presence of 12 mM glucose and absence of serum for 48 h, and apoptosis was assessed by quantification of caspase 3/7 activities. Data are means + SEM, n = 3. *p<0.05 and **p<0.01 compared to the control samples at 1 mM glucose. ^#^ *p*<0.05 and ^##^ *p*<0.01 compared to the control samples at 12 mM glucose. (**B–D**) Effects of CaMK4 and CREB on MIN6 β-cell apoptosis. 24 h after MIN6 β-cells had been transiently transfected with plasmids as shown, they were maintained in culture for a further 48 h in the absence of serum (B–D) or presence of 10% FBS (D) and caspase-3/7 activities were quantified. Data are means + SEM, n = 3–5. *p<0.05, **p<0.01, ***p<0.001 compared to the control samples. ^##^ *p*<0.01 compared to the Δ^K75E^CaMK4 or ΔCaMK4 transfectant cells, as appropriate.

CREB is known to promote β-cell survival [34] but it is not known if this is downstream of CaMK4 activation, so we further investigated the effects of KN62, ΔCaMK4, Δ^K75E^CaMK4, CREB_WT_, CREB_M1_ and CREB_DIEDLM_ on MIN6 β-cell apoptosis in the absence of serum. As seen in Figure 4A, β-cell apoptosis was highest at 1 mM glucose, and 2.5 and 12 mM glucose decreased caspase-3 and -7 activities in a concentration-dependent manner. However, the protective effect of 12 mM glucose was significantly reduced in the presence of 20 µM (*p*<0.05) and 50µM KN62 (*p*<0.01) suggesting that it is also regulated by calcium/calmodulin-dependent kinases.

A protective role for CaMK4 was demonstrated following expression of constitutively active ΔCaMK4, which led to a significant (*p*<0.01) reduction in caspase-3 and -7 activities to approximately 60% of the apoptosis detected in pcDNA3.1 transfected cells (Con; Figure 4B). An anti-apoptotic effect of CREB was evident from the significant (*p*<0.05) reduction in caspase-3 and -7 activities of MIN6 β-cells transiently expressing constitutively active CREB_DIEDLM_, but CREB_WT_ overexpression did not reduce apoptosis and was less effective than CaMK4 alone when co-expressed with ΔCaMK4 (Figure 4B). However, expression of the dominant negative form of CREB (CREB_M1_) resulted in increased apoptosis at 4 mM glucose (*p*<0.01) and abolished the capacity of ΔCaMK4 to inhibit MIN6 β-cell caspase-3/7 activities (*p*<0.01, Figure 4C), suggesting that CREB mediates the anti-apoptotic effect of CaMK4. Consistent with this, Δ^K75E^CaMK4 expression was associated with a significant increase in caspase-3 and -7 activity levels (*p*<0.01) and co-expression of CREB_DIEDLM_ abolished this effect (*p*<0.01, Figure 4C). Figure 4D demonstrates the effects of CREB_M1_ and Δ^K75E^CaMK4 expression on caspase 3/7 activities of non-stressed MIN6 β-cells that had been maintained in serum-containing medium for 48 hours, compared to data obtained with serum-starved cells. Consistent with the data shown in Figure 1, there was a significant increase (*p*<0.001) in apoptosis in serum-deprived cells, and cells expressing dominant negative CaMK4 or CREB showed significantly higher (*p*<0.001) levels of apoptosis irrespective of whether the cells had been incubated in the absence or presence of FBS.

## Discussion

Progressive loss of insulin sensitivity and β-cell function are the two main factors that contribute to the development of T2DM [1], [3], [5], [39]. However, although the majority of individuals with T2DM are mildly or overtly obese, most obese people and pregnant women, who can have severe insulin resistance, do not develop T2DM [2], [3], [6], [10], [40]. The precise physiological explanations for this are not yet entirely understood, but several reports suggest that this is due to a compensatory mechanism that involves increased β-cell mass [2], [3], [6]–[10], [41]. Accordingly, finding novel molecular targets to expand β-cell mass through the stimulation of proliferation and/or survival has the capacity to provide therapeutic benefit, and the data presented in this paper suggest that CaMK4 is one of these molecules.

CaMK4 is a multifunctional enzyme that has been extensively studied in neurons, where it stimulates calcium-dependent, CREB-mediated stimulation of dendritic growth [25] and cell survival [26]–[29]. CaMK4 is also expressed by islet β-cells [24], [30], [31], and previous reports indicate that it plays an important role in the cellular mechanisms by which insulin gene expression is regulated [24], [30]. More recently, it has been shown that glucose stimulates β-cell IRS-2 gene expression [36], [37] and we demonstrated that this effect is dependent upon the activation of the islet CaMK4-CREB signalling pathway [31]. Deletion of IRS-2 in mice results in the development of T2DM, in part due to reduced β-cell mass [16], [17], and CREB-induced IRS-2 expression prevents diabetes by promoting β-cell survival [18], [34]. Thus, while earlier reports have indicated the importance of CREB and IRS-2 in β-cell mass regulation, this is the first study to have determined whether CaMK4 is upstream of CREB and IRS-2 to regulate β-cell proliferation and apoptosis.

Our demonstration that knocking down IRS-2 expression in MIN6 β-cells was associated with a complete inhibition of glucose-stimulated proliferation and with a significant reduction in β-cell survival, are consistent with a crucial role for IRS-2 in the regulation of β-cell mass expansion. In addition, experiments in which caspase 3/7 activities were quantified after MIN6 β-cells had been maintained in the presence of 10% FBS throughout indicated that IRS-2 knockdown by itself was sufficient to increase β-cell apoptosis without the additional stress of 48 hours of serum withdrawal. Moreover, the importance of CaMK4 in the regulation of β-cell proliferation and apoptosis was established by our results demonstrating that both stable and transient expression of the constitutively active form of CaMK4 (ΔCaMK4) promoted MIN6 β-cell proliferation, whereas apoptosis was minimal when ΔCaMK4 was expressed. This was also confirmed by our observations that the stimulatory effect of glucose on BrdU incorporation into newly synthesised DNA and MIN6 β-cell survival were significantly reduced by the use of the kinase dead form of CaMK4 (Δ^K75E^CaMK4). The observation that MIN6 β-cells compensated for introduction of Δ^K75E^CaMK4 with increased expression of the endogenous kinase to maintain proliferation further confirms that CaMK4 plays a critical role in β-cell division.

Our data are also consistent with CREB activation being essential for the anti-apoptotic and proliferative effects of CaMK4. As for ΔCaMK4, expression of the constitutively active form of CREB (CREB_DIEDLM_) resulted in significant reductions in caspase-3/7 activities and stimulation of MIN6 β-cell division. More importantly, co-expression of the dominant negative form of CREB (CREB_M1_) with ΔCaMK4 abolished the protective and proliferative effects of CaMK4, and the negative effects of Δ^K75E^CaMK4 expression on apoptosis and proliferation were rescued by co-transfecting the cells with CREB_DIEDLM_. However, CREB (_WT_) overexpression did not potentiate the survival and proliferative actions of ΔCaMK4, suggesting that endogenous CREB is not rate limiting in these CaMK4 effects. The increased apoptosis obtained following expression of dominant negative CREB_M1_ or Δ^K75E^CaMK4 was observed in cells that had been maintained for 48 hours in the presence of 10% FBS as well as in serum-starved cells, indicating that impeding transduction through the CaMK4/CREB signalling cascade can promote apoptosis in non-stressed β-cells, as well as in those in which apoptosis had already been induced by serum depletion.

**Figure 5:**
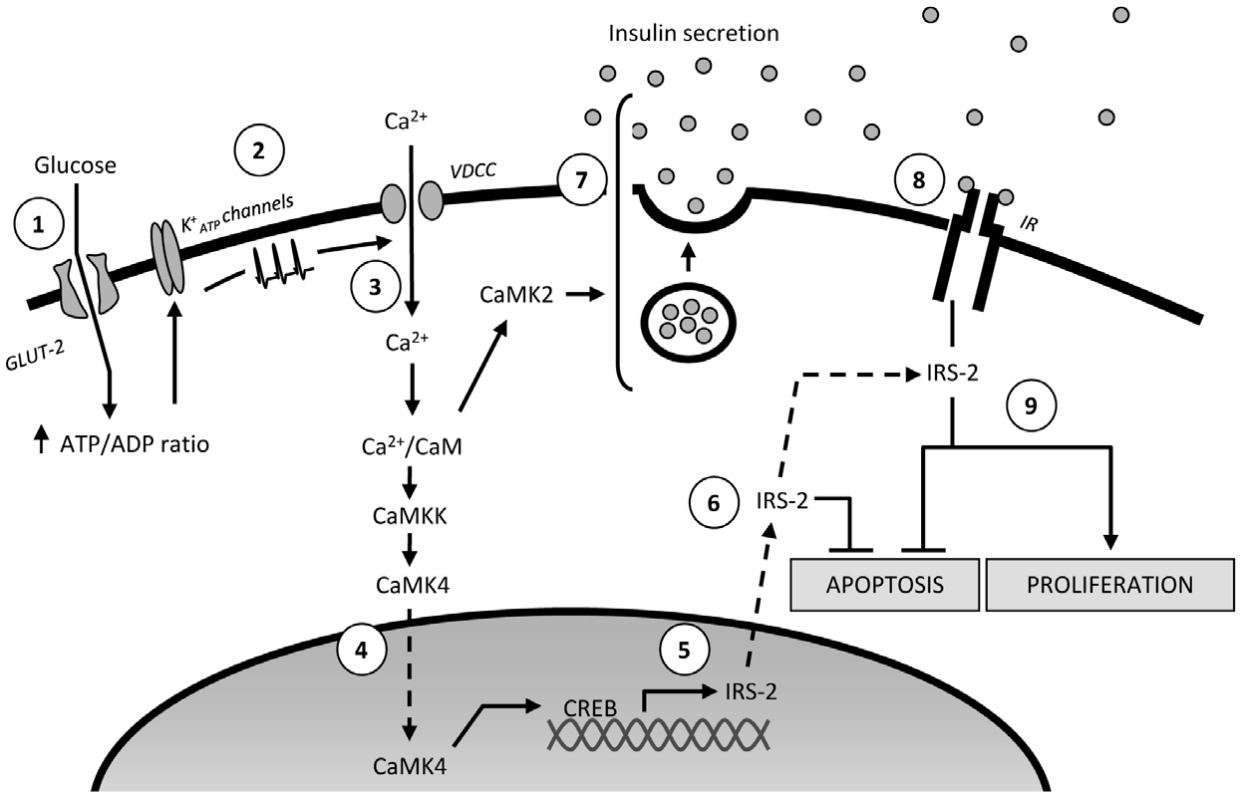
Schematic showing the role of the CaMK4-CREB pathway in the regulation of β-cell mass. Glucose enters β-cells via GLUT-2 transporters, and its metabolism generates an increase in the intracellular ATP/ADP ratio (1), which leads to closure of ATP-dependent potassium (K^+^_ATP_) channels, generating plasma membrane depolarisation (2). Calcium enters through voltage-dependent calcium channels and binds to calmodulin (CaM), and this complex activates CaMKKs/CaMKs signalling cascades (3). Upon its activation, CaMKK phosphorylates CaMK4, which translocates to the nucleus and phosphorylates the cAMP response element binding protein (CREB) on serine 133 (4). This phosphorylation is responsible for CREB activation which, in turn, binds to the cAMP response element (CRE) on the IRS-2 promoter to increase IRS-2 gene expression (5). IRS-2 maintains β-cell mass by stimulating proliferation and inhibiting apoptosis (6). In addition to this CaMK4/CREB-mediated pathway, calcium/calmodulin-induced activation of CaMK2 and CaMK4 stimulate insulin secretion and insulin gene expression (7) and this results in an autocrine/paracrine activation of the β-cell insulin signalling cascade (8), which ultimately increases β-cell proliferation and reduces apoptosis, at least in part, in an IRS-2-dependent manner (9).

The data generated in this study allow us to propose a model in which CaMK4 plays a central role in the signalling cascade through which glucose regulates β-cell apoptosis and proliferation (Figure 5). Thus, our observations are consistent with glucose-induced elevations in intracellular Ca^2+^ and the consequent activation of CaMK4 playing a key role in CREB-dependent up-regulation of IRS-2, and maintenance of β-cell mass. Previous data demonstrating CaMK4-stimulated insulin gene expression [24], [30] and CaMK2-induced insulin secretion [42] fit with our model and imply that these events are interconnected *via* autocrine/paracrine activation of the β-cell insulin signalling cascade.

In conclusion, our study indicates that CaMK4 regulates β-cell apoptosis and proliferation in part *via* CREB activation. Our observations suggest that targeting CaMK4 expression and/or activity to increase β-cell mass may be effective in counteracting the progressive development of T2DM.

## Materials and Methods

### Reagents and Plasmids

Plasmids encoding the mouse constitutively active (ΔCaMK4) and human kinase-dead (Δ^K73E^CaMK4) forms of CaMK4 were kindly provided by Prof. A. Ghosh (University of California, San Diego, USA), and pcDNA3.1 plasmids encoding the wild-type (WT), constitutively active (DIEDLM) and dominant negative (M1) forms of CREB were a gift from Prof. D.D. Ginty (Howard Hughes Medical Institute, Maryland USA). Lipofectamine 2000, glucose-free DMEM and fetal bovine serum (FBS) were purchased from Invitrogen (Paisley, UK). G418 and KN62 were from Calbiochem (Nottingham, UK). The mouse monoclonal anti-α-tubulin antibody, culture media, penicillin/streptomycin and L-glutamine were from Sigma Aldrich (Dorset, UK). The rabbit polyclonal anti-IRS-1 and anti-IRS-2 antibodies were from Millipore UK Ltd (Watford, UK) and the mouse monoclonal anti-CaMK4 antibody was from Clontech (Oxford, UK). Horseradish peroxidase (HRP)-conjugated secondary antibodies were from Pierce Biotechnology (Rockford, IL, USA). The siRNA duplexes designed to knock down IRS-2 expression and non-silencing RNAs were obtained from Dharmacon (Northumberland, UK). The nucleofector device was from Amaxa (Köln, Germany). Enhanced chemiluminescent kits for Western blotting were from GE Healthcare (Bucks, UK), colorimetric BrdU cell proliferation ELISA kits were from Roche Diagnostics Ltd (West Sussex, UK) and Caspase Glo-3/7 assay kits were from Promega (Southampton, UK).

### Cell Culture and Transfection

These experiments have been performed on mouse MIN6 β-cells [43] as they are a well-established physiological β-cell model that allows investigation of signalling cascades in the absence of potential confounding inputs from other islet endocrine cells. MIN6 β-cells were maintained in culture at 37°C in DMEM (25 mM glucose) supplemented with 2 mM glutamine, 10% FBS, 100 units/ml penicillin and 100 µg/ml streptomycin. Transient transfection of MIN6 β-cells was achieved by electroporation using a Nucleofector II as previously described, with a transfection efficiency of 60–80% [31]. Pre-treatment with the CaMKs inhibitor KN62 (or DMSO vehicle, 45 minutes) and transient transfection with non-silencing RNA or siRNA duplexes designed to knock down IRS-2 expression were performed before the final adjustment of the glucose concentration. For some experiments, MIN6 β-cells were transfected with plasmids expressing ΔCaMK4 and Δ^K75E^CaMK4 using Lipofectamine 2000 and stably transfected colonies were selected by maintenance in the presence of 800 µg/ml G418. G418-resistant colonies of MIN6 β-cells were expanded and ΔCaMK4 and Δ^K75E^CaMK4 expression was determined by Western blotting. MIN6 β-cells were maintained in culture in the presence of 10% FBS for 24 hours after transient transfections, then for an additional 48 hours in the absence of FBS or the continued presence of 10% FBS, as detailed in the Figure legends.

### Western Blotting

MIN6 β-cell protein extracts (50–75 µg) were separated on 10% polyacrylamide gels and transferred to PVDF membranes, which were incubated for 16 hours with antibodies directed against IRS-1 (1∶166 dilution), IRS-2 (1∶1,000 dilution), CaMK4 (1∶750 dilution) or α-tubulin (1∶5,000 dilution). After 3 washes in Tris buffered saline (pH 7.4) containing 0.05% Tween 20 (TBS-T), the PVDF membranes were incubated for another hour with HRP-coupled anti-rabbit or anti-mouse IgGs as appropriate (1∶5,000 dilution). Binding of secondary antibodies was revealed by chemiluminescence.

### Proliferation Assay

Proliferation was quantified by measuring the levels of BrdU incorporation into newly synthesised MIN6 β-cell DNA, essentially as previously described [33]. Thus, native MIN6 β-cells or those that had been transfected with niRNA or siRNA duplexes or with CaMK4 and/or CREB plasmids were seeded at a density of 15,000 cells per well in 96-well plates and serum- and glucose-deprived for 24 hours. They were then incubated for 48 hours in the presence of 10% FBS and the glucose concentrations as described in the Figure legends, and media were supplemented with 10 µM BrdU for the final 2 hours of incubation. BrdU incorporation was assessed by measuring absorbance at 450 nm after incubation with an anti-BrdU-peroxidase coupled antibody and addition of the peroxidase substrate TMB.

### Apoptosis Assay

Apoptosis was quantified by measuring the MIN6 β-cell caspase-3 and -7 activities by bioluminescence using the Caspase Glo-3/7 assay, essentially as described by the manufacturer. In these experiments 15,000 native MIN6 β-cells or those that had been transfected with niRNA or siRNA duplexes or with CaMK4 and/or CREB plasmids, were maintained in the absence or presence of 10% FBS for 48 hours, under the conditions described in the Figure legends. Caspase 3/7-induced cleavage of a luminogenic substrate was quantified using a luminometer.

### Statistical Analysis

Numerical values are expressed as means ± SEM. Student’s t tests were used for statistical analyses of the differences between two groups, and the statistical significance of differences among multiple groups was determined by ANOVA followed by the Tukey HSD test.
